# Conventional radiography requires a MRI-estimated bone volume loss of 20% to 30% to allow certain detection of bone erosions in rheumatoid arthritis metacarpophalangeal joints

**DOI:** 10.1186/ar1919

**Published:** 2006-03-15

**Authors:** Bo Jannik Ejbjerg, Aage Vestergaard, Søren Jacobsen, Henrik Thomsen, Mikkel Østergaard

**Affiliations:** 1 Department of Rheumatology, Copenhagen University Hospitals at Hvidovre and Gentofte, Kettegaard Allé30, 2650 Hvidovre, Denmark; 2 Department of Radiology, Copenhagen University Hospital at Hvidovre, Kettegaard Allé30, 2650 Hvidovre, Denmark; 3 Department of Rheumatology, Copenhagen University Hospitals at Rigshospitalet, Blegdamsvej 9, 2100 Copenhagen, Denmark; 4 Department of Diagnostic Radiology, Copenhagen University Hospitals at Herlev, Herlev Ringvej 75, 2730 Herlev, Denmark; 5 Department of Rheumatology, Copenhagen University Hospitals at Hvidovre and Herley, Kettegaard Allé30, 2650 Hvidovre, Denmark

## Abstract

The aim of this study was to demonstrate the ability of conventional radiography to detect bone erosions of different sizes in metacarpophalangeal (MCP) joints of rheumatoid arthritis (RA) patients using magnetic resonance imaging (MRI) as the standard reference. A 0.2 T Esaote dedicated extremity MRI unit was used to obtain axial and coronal T1-weighted gradient echo images of the dominant 2nd to 5th MCP joints of 69 RA patients. MR images were obtained and evaluated for bone erosions according to the OMERACT recommendations. Conventional radiographs of the 2nd to 5th MCP joints were obtained in posterior-anterior projection and evaluated for bone erosions. The MRI and radiography readers were blinded to each other's assessments. Grade 1 MRI erosions (1% to 10% of bone volume eroded) were detected by radiography in 20%, 4%, 7% and 13% in the 2nd, 3rd, 4th and 5th MCP joint, respectively. Corresponding results for grade 2 erosions (11% to 20% of bone volume eroded) were 42%, 10%, 60% and 24%, and for grade 3 erosions (21% to 30% of bone volume eroded) 75%, 67%, 75% and 100%. All grade 4 (and above) erosions were detected on radiographs. Conventional radiography required a MRI-estimated bone erosion volume of 20% to 30% to allow a certain detection, indicating that MRI is a better method for detection and grading of minor erosive changes in RA MCP joints.

## Introduction

Conventional radiography offers information about destructive joint changes and has been the mainstay in diagnostic imaging in inflammatory arthropathies for decades. Radiographic erosion and/or periarticular osteopenia is one of the American College of Rheumatology 1987 revised criteria for the classification of rheumatoid arthritis (RA) [1]. Conventional radiography is the recommended method for monitoring progression of structural joint changes in the routine management of RA patients as well as in clinical trials [2].

It has a lower sensitivity than MRI for bone erosions [3], although the relative difference, that is, how large MRI erosions need to be before they are detectable on conventional radiography, is not known.

The objective of this study was, with MRI as the standard reference, to demonstrate the ability of conventional radiography to detect bone erosions of different sizes in RA metacarpophalangeal (MCP) joints.

## Materials and methods

Sixty-nine rheumatoid arthritis patients, 57 female and 12 male, fulfilling the American College of Rheumatology 1987 revised diagnostic criteria [1] were enrolled in the study. The median age and disease duration was 51 years (24 to 85 years) and 5 years (2 months to 37 years), respectively. Seventy-three percent of the patients were IgM rheumatoid factor positive. Local ethics committee approval was obtained prior to study initiation, and signed informed consent was obtained from all patients.

### Magnetic resonance imaging

MRI of the 2nd to 5th MCP joints was performed on a 0.2 Tesla dedicated extremity MRI unit (Artoscan, Esaote Biomedica, Genova, Italy) equipped with a dual phased array wrist coil. All MRI examinations were carried out using a T1 weighted three-dimensional gradient echo sequence with subsequent multiplanar reconstruction. The scanning parameters were: repetition time 30 ms, echo time 12 ms, slice thickness 1 mm, field of view 140 mm × 140 mm × 80 mm, matrix 192 × 160 × 80, number of acquisitions 1, flip angle 65°, voxel size 0.54 × 0.54, scanning time 6.25 minutes.

MCP joint bones (the metacarpal head and the phalangeal base) were assessed separately for erosions according to the outcome measures in rheumatology (OMERACT) recommendations [4]. By this method, erosions are scored on a scale from 0 to 10 based on the proportion of eroded bone compared to the 'assessed bone volume' judged on all available images: 0 = no erosion; 1 = 1% to 10% of the bone eroded; 2 = 11% to 20% of the bone eroded; 3 = 21% to 30% of the bone eroded, and so on. In long bones, 'the assessed bone volume' covers the area from the articular surface to a depth of 1 cm. Merge eFilm™(Milwaukee, Wisconsin, USA) workstation, a commercially available software package, was used for the readings of the MRI images. This software enables digital image viewing and provides the reader with advanced features of image viewing, for example, window/level settings, zooming, and three-dimensional localizing for accurate identification of specific lesions in perpendicular planes. All MR images were evaluated by the same rheumatologist (BE), who was blinded to the results of the assessment of the radiographs.

### Conventional radiography

Conventional radiographs of the 2nd to 5th MCP joints were obtained in the posterior-anterior projection. All radiographs were evaluated by the same experienced musculoskeletal radiologist (AaV), who was blinded to the results of the MRI assessment.

### Statistical analysis

The sensitivity of conventional radiography for detection of MRI erosions of different sizes was calculated.

## Results

**Figure 1:**
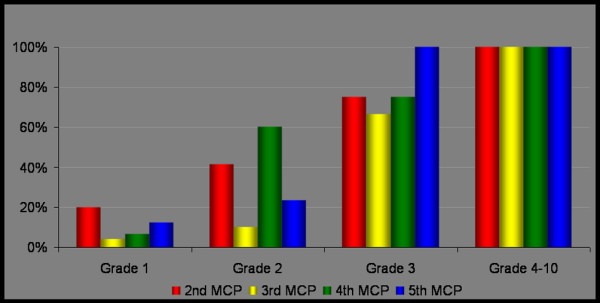
The sensitivity of conventional radiography for detection of magnetic resonance imaging erosions. An OMERACT grade 1 erosion comprises 1% to 10% of the 'assessed bone volume', whereas OMERACT grade 2 and 3 erosions comprise 11% to 20% and 21% to 30%, respectively (see Materials and methods for a detailed description). OMERACT grade 4 to 10 erosions are erosions that occupy more than 31% of the 'assessed bone volume'. MCP, metacarpophalangeal joint.

In total, 276 MCP joints (552 bones) were assessed and 123 MRI erosions were detected. We observed a preponderance of the MRI erosions towards the radial MCP joints with 43 and 34 erosions in the 2nd and 3rd MCP joints, respectively. Twenty-three erosions were detected in the 4th MCP as well as in the 5th MCP joint. Figure 1 depicts the sensitivity of conventional radiography for detection of the erosive changes observed on MRI. Small MRI erosions (grades 1 and 2), comprising 1% to 20% of the bone volume, were most often not detected on conventional radiography. Grade 3 MRI erosions (21% to 30% of the bone volume) were always detected in the 5th MCP joints. However, only 75%, 67% and 75% of the grade 3 MRI erosions were detected on conventional radiography in the 2nd, 3rd and 4th MCP joints, respectively. Conversely, all erosions of grade 4 and above (≥31%) were identified on conventional radiography. Three erosions were identified on conventional radiography but not on MRI.

## Discussion

In this study, minor erosive changes (≤30% of the assessed bone volume (OMERACT grades 1 to 3)) as judged on MRI in rheumatoid MCP joints were most often not detected on conventional radiography. In contrast, MRI erosions exceeding OMERACT grade 3 (≥31% of the assessed bone volume) were always detected on conventional radiography.

The OMERACT RA MRI scoring system (RAMRIS) was chosen for the assessment of the destructive joint changes. The RAMRIS scoring system is based on iterative scoring exercises and subsequent consensus among an international group of MRI experts and includes definitions of MRI erosions and describes in detail the grading of erosions [4]. This scoring system does not, however, take into account where an erosion is located, only whether it is in the correct juxtaarticular area and complies with the definitions or not.

The fact that some of the smaller (grades 1 to 3) MRI erosions are sometimes detected may to some extent be explained by the location of the erosion, for example, in areas with low bone thickness, thus dimishing projectional superimposition artefacts. In other words, conventional radiography is probably more sensitive in certain areas. This aspect is further supported by our results as grade 3 erosions were always detected in the 5th MCP joint. In even smaller bones, for example, the 5th metatarsophalangeal joint, conventional radiography may perform even better, as suggested by Forslind and colleagues [5].

Destructive joint damage judged on conventional radiography occurs within the first years of RA [6] and early detection of erosions is closely related to poor outcome [7]. The treatment strategy has changed because increasing data suggest that prompt disease control improves long term outcome. Accordingly, there is a growing need for tools for early diagnosis, separation of responders from non-responders and for prediction of disease course. MRI has gained increasing interest in recent years because it has been shown to detect erosive changes in RA earlier than conventional radiography [3] and to predict later erosive development in early as well as more established disease [8,9]. Recently, MRI has also been shown to be more sensitive to RA destructive joint changes than conventional radiography [10]. Increasing evidence indicates that erosions detected by MRI are real erosions: Perry and colleagues [11] compared computed tomography and MRI in nine rheumatoid wrists and reported an 87% concordance between CT and MRI and only 4% of the erosions detected on MRI were not confirmed by CT. Furthermore, Ostendorf and colleagues [12] reported that MCP joint bone erosions observed on MRI represent real bone pathology as judged on miniarthroscopy. Our group reported recently that low field dedicated MRI, as used in the present study, is highly sensitive and specific for detection as well as grading of bone erosions when compared to standard high field MRI of wrist and MCP joints [13]. The present study, using exactly the same MRI sequences, supports these observations. It should be emphasized that various artefacts and pitfalls have to be considered in the interpretation of MR images [14].

We do find it surprising that up to 30% of a MCP joint bone must be eroded before the erosion is detected on conventional radiography. Although the MRI scoring system has not yet been tested against computed tomography, which can be considered a standard reference for detection of loss of calcified tissue, the available data suggest a high agreement between MRI and computed tomography erosions [11] as well as in radiographically non-eroded areas [15]. Furthermore, the scoring system is well validated and the present MR image reader (BE) has previously demonstrated a high inter- and intra-observer reliability [10,16]. Overall, the results of the cited studies suggest that MRI observations of erosions are true and reliable.

## Conclusion

Our results put a question mark against conventional radiography as being the most competent imaging modality in RA and indicates that MRI is better suited for detection and grading of minor erosive joint changes in RA.

## Abbreviations

MCP = metacarpophalangeal; MRI = magnetic resonance imaging; OMERACT = outcome measures in rheumatology; RA = rheumatoid arthritis; RAMRIS = (OMERACT) RA MRI Scoring System.

## Competing interests

The authors declare that they have no competing interests.

## Authors' contributions

BE: study design, acquisition of data, analysis of data and interpretation of data and writing the manuscript. AaV: acquisition and analysis of data. SJ: interpretation of data and drafting the manuscript. HST: acquisition and analysis of data. MØ: study design, analysis of data and interpretation of data and writing the manuscript.
